# Isolated *Sporothrix schenckii* Monoarthritis

**DOI:** 10.1155/2018/9037657

**Published:** 2018-06-12

**Authors:** Aram Barbaryan, Wissam El Atrouni, Stefania Bailuc, Matthew W. Jones, Maharshi Bhakta, Khaldoun Haj Mahmoud, Aibek E. Mirrakhimov

**Affiliations:** ^1^Department of Internal Medicine, Division of General, Geriatric & Hospital Medicine, University of Kansas Health System, Kansas City, KS, USA; ^2^Division of Infectious Diseases, Department of Internal Medicine, University of Kansas Health System, Kansas City, KS, USA; ^3^Department of Medicine, University of Kentucky, Lexington, KY, USA

## Abstract

*Sporothrix schenkii sensu lato* is a rare cause of arthritis. Its course is indolent with lack of constitutional symptoms resulting in delayed presentation and diagnosis. It is a dimorphic fungus found ubiquitously in sphagnum moss, decaying vegetation, soil, and hay. Inoculation of dirt into the skin and soft tissues and, in rare instances, inhalation of aerosolized conidia from soil and plants can lead to infection. Subacute and chronic involvement of skin and subcutaneous tissues is the most common manifestation of sporotrichosis in immunocompetent hosts. In patients with underlying risk factors (HIV, alcoholism, diabetes mellitus, organ transplant patients, immunosuppressive medications, steroids, and malignancies), it can often have disseminated visceral, osteoarticular, meningeal, and pulmonary involvement. Sporothrical arthritis most commonly infects knee joint followed by hand and wrist joints. A culture of *Sporothrix schenkii sensu lato* is the gold standard for the diagnosis of sporotrichosis. Itraconazole is the drug of choice for osteoarticular sporotrichosis. We present a case of sporotrichal arthritis in a patient without skin or lymph node involvement who underwent treatment with itraconazole resulting in resolution of his symptoms.

## 1. Case Presentation

**Figure 1:**
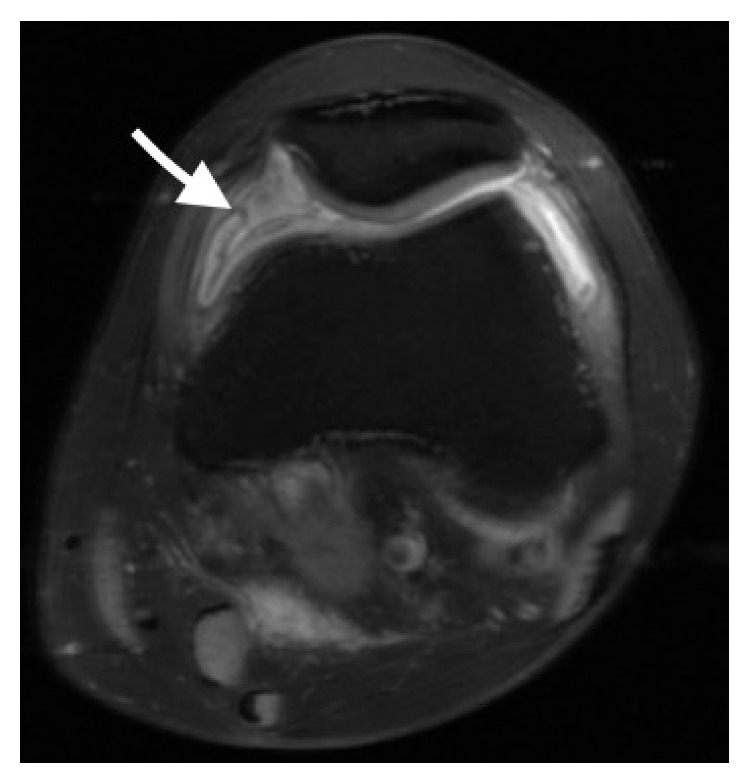
MRI (axial view) of left knee showing synovial thickening (arrow).

A 33-year-old male taxi driver with past medical history significant only for alcoholism, presented to his family physician's office with the chief complaint of left knee pain and swelling for nine months. He denied any specific injury, puncture wound, or any systemic symptoms. He denied contact with animals. Examination of his left knee demonstrated a moderate-to-severe effusion. He was not tender to palpation across his bony prominences. He had no medial or lateral joint line tenderness. He had a range of motion that was full and symmetric with that of the contralateral side. He had a negative McMurray's sign. His knee was stable to ligamentous examination. There was no erythema or lymphadenopathy. X-ray of the left knee showed a joint effusion. MRI of his left knee also demonstrated a joint effusion with a popliteal cyst and synovial thickening (Figure 1).

**Figure 2:**
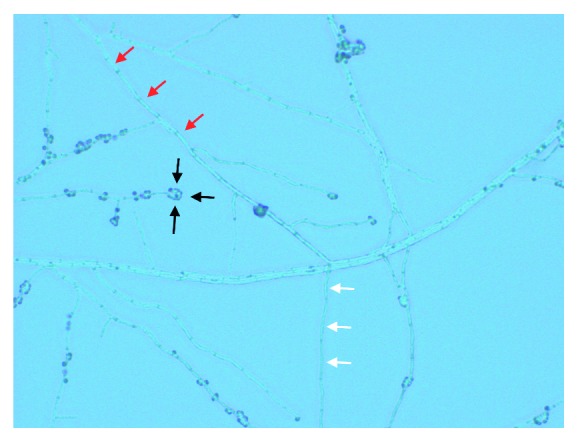
A hyphal form of *Sporothrix schenkii* from synovial fluid aspirate cultured on Sabouraud agar. Narrow branching hyphae (red arrows) giving rise to slender conidiophores (white arrows) at right angles. The apex of conidiophores is covered with tear-shaped conidia in a rosette-like fashion (black arrows). Single conidia can also be formed along the hyphae.

He subsequently underwent aspiration of the effusion in the office. Synovial fluid was cloudy yellow and blood-tinged. Fluid analysis showed the following: RBC: 50,200 cells/mcL, WBC: 4900 cells/mcL (ref range < 150/mcL), PMN: 34% (ref range 0–25%), lymphocytes: 54%, and monocyte: 10%; no crystals were observed, and pathological exam was consistent with hemorrhagic fluid with acute and chronic inflammation. Bacterial culture did not demonstrate any growth, but fungal cultures grew branching narrow hyphae with septations and conidia in a bouquet-like appearance leading to a presumptive diagnosis of *Sporothrix schenkii* (Figure 2). The patient was admitted to the University of Kansas Hospital for further management.

The patient underwent diagnostic left knee arthroscopy, irrigation, debridement, and major synovectomy. There was no internal derangement of the knee. There was no chondral injury. He had no evidence of a medial-lateral meniscus tear. His anterior cruciate ligament was intact. There was significant synovial thickening throughout. Blood counts, liver biochemistry, coagulation parameters, and renal and thyroid functions were normal except for elevated liver function tests consistent with his history of alcohol use. ESR and CRP were within normal limits, HIV test was negative, and immunodeficiency workup (humoral immunodeficiency, phagocytic disorders, and T-cell immunodeficiencies) was unrevealing. Abdominal CT done to evaluate for disseminated disease showed marked diffuse hepatic steatosis. After the debridement, the patient was started on oral itraconazole 200 mg twice daily with a plan to treat for 12 months.

Synovial biopsies obtained during debridement from the suprapatellar pouch were sent for both microbiology and pathology. Surgical cultures on Sabouraud dextrose agar grew *Sporothrix schenkii* within four days confirming the initial diagnosis. The identification was confirmed by matrix-assisted laser desorption/ionization time-of-flight mass spectrometry—MALDI-TOF MS (Bruker Daltonics Biotyper Microflex LT).

**Figure 3:**
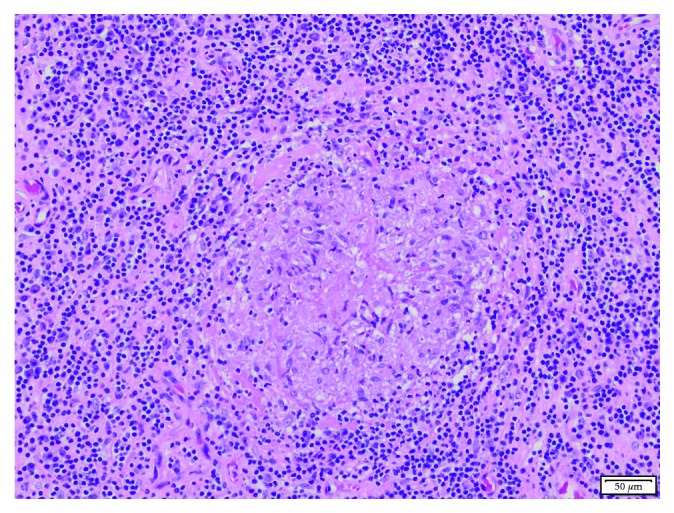
Histopathologic features of excisional biopsy. H/E-stained sections demonstrate mixed chronic inflammation composed of plasma cells and lymphocytes. Scattered granulomas are present with central necrosis (×200).

All other microbiological investigations of effusion samples including routine bacterial cultures, acid-fast bacilli (AFB) cultures, were ultimately negative for any growth. Blood cultures multiple times did not reveal any growth. Pathology of the synovial tissue showed prominent mixed chronic inflammation composed of plasma cells and lymphocytes. There were scattered granulomas with central necrosis. Fite's acid-fast staining was negative for acid-fast bacilli. GMS stain was negative for fungal organisms (Figure 3).

Antifungal susceptibility assays were performed by the broth microdilution method according to the guidelines recommended by the Clinical Laboratory Standards Institute (CLSI): documents M38-A2 (CLSI, 2002b) (Table 1).

During his subsequent follow-up visits, he reported that all his symptoms (knee swelling and pain) had improved with itraconazole, but unfortunately, he continued to drink excessive amounts of alcohol.

## 2. Discussion

Sporotrichosis was first diagnosed by Schenck in 1896 at Johns Hopkins Hospital from a 36-year-old male patient presenting with right hand and arm lesions [1]. *Sporothrix schenkii sensu lato* is a dimorphic fungus. At 25–30°C (in the environment or the laboratory), the organism grows as a mold. The hyphae are narrow with septations and branching with tapering conidiophores rising at right angles. The apex of conidiophores carries many tear-shaped and round conidia in a bouquet-like appearance forming rosette-like structures. In vivo at 35–37°C, *Sporothrix schenkii* creates yeast-like colonies comprising round, oval, or fusiform budding cells [2]. *Sporothrix schenkii sensu lato* is a complex comprising four related species: *S. schenckii sensu stricto, S. brasiliensis, S. globosa, and S. lurei. S. schenckii sensu stricto* is the most common species associated with human diseases [3, 4]. In our case, no molecular identification was made; therefore, it is necessary to call it *Sporothrix schenkii sensu lato.*

The organism grows densely in sphagnum moss, decaying vegetation, soil, and hay. People involved in outdoor activities are at high risks like gardeners, nursery workers, farmers, and miners. Accidents that lead to inoculation of soil into the skin and soft tissues can lead to infection. History of trauma is not always present [5]. As in this case, probably the inoculation happened through microscopic breaks in the skin or through small injuries the patient did not remember. There are some cases when *Sporothrix schenkii sensu lato* caused disease through intact skin when inoculum was large. In those cases, the infection was acquired through contact with cats [6, 7]. *Sporothrix schenkii sensu lato* can also be transmitted through animal bites and scratches. In cases of pulmonary sporotrichosis, the mode of transmission is through inhalation of aerosolized conidia from soil and vegetations [5].

Subacute and chronic involvement of the skin and subcutaneous tissues is the most common manifestation of sporotrichosis in immunocompetent hosts. The inoculum load and virulence factors of the strain, immune status of the patient, as well as the depth of inoculation determine the various clinical forms of sporotrichosis [8]. In patients with underlying risk factors (HIV, alcoholism, diabetes mellitus, organ transplant recipients, malignancies, and use of immunosuppressive medications or steroids), it can often have disseminated visceral, osteoarticular, meningeal, and pulmonary involvement [5, 9, 10]. Excessive amounts of alcohol consumption is an important risk factor. Bayer et al. described 44 cases of sporotrichal arthritis, of which 38% were alcoholics. In another report by Howell et al., 10 out of 13 cases of sporotrichal arthritis had a history of significant alcohol intake [11, 12]. Osteoarticular disease is rare representing 3-4% of cases, most often affecting patients with alcoholism as is the case with our patient [11–14]. It is the most common extracutaneous form of sporotrichosis [15]. Its course is indolent with lack of constitutional symptoms resulting in delayed presentation and diagnosis [11]. Osteoarticular form can either follow skin inoculation or results from hematogenous spread from the lungs. Isolated involvement of joints without skin lesions is rare. Septic arthritis is more common than osteomyelitis. Sporotrichosis most commonly infects the knee joint followed by hand and wrist joints [11, 13]. It has a propensity to affect small joints in the hands and the wrist in contrast to other fungal arthritides (*Candida* and *Coccidioides*) [11, 13, 16]. It can be monoarticular (more common in immunocompetent hosts) or polyarticular. Other manifestations of osteoarticular sporotrichosis include granulomatous tenosynovitis and bursitis [8, 17, 18].

Patients with sporotrichal arthritis usually present with joint swelling, tenderness, restricted range of motion, and stiffness. Only a minority of patients have positive constitutional signs, fever, and leukocytosis. Elevated erythrocyte sedimentation rate is common [19]. The course is indolent with an average time to diagnosis of 17 months. The most common abnormality on X-ray is osteoporosis of contiguous bony surfaces followed by soft tissue and parasynovial swelling, cartilage erosion, and punched-out bony lesions [11].

Culture is the gold standard for the diagnosis of sporotrichosis. It consists of isolating the organism from a clinical specimen. Sabouraud dextrose agar is the medium of choice. Within days to weeks (in 89% of cases within eight days), hyphal growth appears at room temperature with characteristic appearance of conidia (bouquet-like or rosette-like structures) resulting in the presumptive identification of *Sporothrix schenkii sensu lato* [5, 20]. For definitive diagnosis, conversion to the yeast form is required by subculturing the organism on special media like brain heart infusion agar, chocolate agar, or blood agar at 35 to 37°C for 5 to 7 days [8, 21]. Another method of identification and differentiation of closely related *Sporothrix* species is by ribosomal protein analysis (obtained directly from fungal cells) using matrix-assisted laser desorption/ionization time-of-flight mass spectrometry—MALDI-TOF MS [22].

Histopathologic examination usually reveals mixed granulomatous and pyogenic inflammation with multinucleated giant cells. The typical lesion is noncaseating granulomatous synovitis, but in some cases, caseating granulomas are seen mimicking tuberculous arthritis. The organisms are frequently present in small numbers, so clinical specimens from patients infected with *Sporothrix schenkii sensu lato* often demonstrates no fungal elements. Either 10% potassium hydroxide, fluorescent antibody staining, Gram stain, or Giemsa stain can be used, but the sensitivity is low. If yeast forms are present, they are usually round, oval, cigar-shaped, or fusiform budding cells [5, 11, 23].

Microdilution test proposed by Clinical Laboratory Standards Institute (CLSI) is the most commonly used susceptibility method standardized for *Sporothrix schenkii sensu lato.* Even though no MIC breakpoints for dimorphic fungi have been established, isolates with MIC < 1 g/ml can be considered susceptible for analytical purposes only [24].

Itraconazole (200 mg twice daily by mouth for at least 12 months) is the drug of choice for osteoarticular sporotrichosis according to the most recent guidelines [25]. Osteoarticular sporotrichosis has a less favorable response rate to itraconazole compared with cutaneous or lymphocutaneous sporotrichosis [26–30]. All forms of sporotrichosis respond to fluconazole suboptimally [29, 30]. Amphotericin B can be used in extensive or itraconazole-unresponsive disease with similar efficacy but with more side effects [11]. Surgical debridement alone is not effective, and it is not commonly used. In most of the cases, surgical therapy was performed for diagnostic purposes as in our case [11, 25].

The rarity of isolated (without skin and lymph node) involvement of joints in sporotrichosis as well as the lack of yeast forms in biopsy specimens results in a delay in diagnosis and subsequently in suboptimal treatment leading to permanent disability and high morbidity. Sporotrichal arthritis should be included in the differential diagnosis of chronic monoarthritis or polyarthritis in patients with high-risk factors and occupation.

## Figures and Tables

**Table 1:** Results of antifungal susceptibility testing.

Drugs	Results (*μ*g/ml)
Amphotericin B	1
Fluconazole	>64
Itraconazole	1
Posaconazole	0.5
Voriconazole	>16
Terbinafine	0.008
